# Influence of Experimental Parameters on Fatigue Crack Growth and Heat Build-Up in Rubber

**DOI:** 10.3390/ma6125502

**Published:** 2013-11-27

**Authors:** Franziska Stadlbauer, Thomas Koch, Vasiliki-Maria Archodoulaki, Florian Planitzer, Wolfgang Fidi, Armin Holzner

**Affiliations:** 1 Institute of Materials Science and Technology, Vienna University of Technology, Favoritenstraße 9-11, Vienna 1040, Austria; E-Mails: thomas.koch@tuwien.ac.at (T.K.); vasiliki-maria.archodoulaki@tuwien.ac.at (V.-M.A.); 2 Semperit Technische Produkte Gesellschaft m.b.H., Triester Bundesstraße 26, Wimpassing 2632, Austria; E-Mails: florian.planitzer@semperit.at (F.P.); wolfgang.fidi@semperit.at (W.F.); armin.holzner@semperit.at (A.H.)

**Keywords:** fatigue, crack growth, rubber, sample geometry, influence factor, frequency, amplitude ratio, loading waveform, heat build-up

## Abstract

Loading parameters (frequency, amplitude ratio and waveform) are varied to determine their influence on fatigue crack growth in rubber. Up to three different rubber blends are investigated: one actual engineering material and two model materials. Fatigue crack growth curves and strain distributions of pure shear and faint waist pure shear samples are compared for a model material. Fatigue behavior is studied for three different frequencies (1 Hz, 3 Hz and 5 Hz). Amplitude ratio appears to be another important influence factor concerning fatigue crack growth in rubber. The beneficial effect of positive amplitude ratios (tensional loading conditions) is shown for different materials. However, fatigue crack growth is considerably increased for negative amplitude ratios (tensional-compressional loading conditions). Furthermore, the influence of the waveform is determined for three different waveform shapes. One is sinusoidal, and two have a square shape, including dwell periods and sinusoidal slopes. Special focus lies on heat build-up, which is substantial, especially for large loads, high frequencies and/or highly filled rubber blends. Plateau temperatures are determined for various loading conditions and rubber blends. A very simple linear relationship with dissipated energy per time and unit area is obtained. Results gathered with dynamic mechanical analyses show, likewise, a linear trend, but the heat build-up is very small, due to different sample geometries.

## 1. Introduction

For technical rubber products, it is essential to estimate their lifetime before usage. In order to do so, failure processes have to be studied. Failure in rubber articles mainly happens due to fatigue, which includes repeated loads smaller than the strain at break. Two approaches to determine the fatigue life of rubber have been established: the crack nucleation approach and the crack growth approach [1]. The first one is based on the assumption that fatigue life depends on the history of a certain quantity (stress, strain or strain energy density) at a point in the material. The second approach deals with fatigue crack growth from preexisting flaws and estimates total fatigue life from fatigue crack growth (FCG) curves [1].

The current study focuses on the crack growth approach and on the FCG curves, which form the experimental output of fatigue analyses within this approach. FCG curves show the crack growth rate in dependence of a loading parameter. As a loading parameter, mainly the tearing energy (also called the energy release rate) is used, which was established by Rivlin and Thomas in 1953 [2]. In various studies [3,4], it was found that FCG curves are independent of sample geometry. This study focuses on pure shear geometries, which have the advantage that the tearing energy is independent of crack length. Furthermore, a broad crack length range can be investigated, due to the small height-to-length ratio. In [5], we compared “flat” pure shear sample geometries with “waisted” ones, and experimental advantages and disadvantages were identified for both. In the current study, this comparison is resumed for another rubber blend.

The total fatigue life of rubber products can be estimated from fatigue crack growth measurements performed in the laboratory [3,4]. In order to obtain reliable results, it is necessary to perform those measurements under loading conditions that correspond well enough to reality [6,7]. Often, loading conditions cannot be directly adopted from reality due to machine limits, exceeding measurement durations, *etc.* The fatigue behavior of rubber strongly depends on various factors that include loading conditions, material parameters and surrounding environmental conditions. For rubber, it is difficult to derive generalities, but by studying the influence of such parameters on fatigue, one can get an idea of the factors that can be adapted without losing reference to reality.

In this study, loading conditions are chosen in relation to the real occurring situation of escalator handrails. These are tested on certain test facilities with bending radii corresponding to reality, but with an accelerated testing speed. That is why investigations focus attention on tension-compression loading conditions according to those bending radii. Fatigue crack growth in pure shear rubber samples has been rarely studied under these conditions, although they occur in various rubber articles. However, also, other loading parameters (frequency and waveform) are investigated, and tension-compressional loads are compared to pure tensional loads. Usually, studies are performed only for model materials that are scientifically important, but not technically relevant. Hence, the current study includes two model materials, as well as an engineering material that is actually used in technical products. These are rubber blends of natural rubber (NR), butadiene rubber (BR) and styrene butadiene rubber (SBR).

Due to cyclic loads, rubber shows a heat build-up if heat generation exceeds heat transfer to surroundings [8,9]. Especially for highly filled rubber blends, for large loads and/or high frequencies, this temperature rise can be significant [6,10]. This heating is frequently neglected or frequencies are chosen in a way that heat build-up does not play a role. However, in reality, frequencies are often quite large. It is important to consider also larger frequencies and the associated heat build-up, because it can add thermal failure to mechanical failure.

In Section 2, the results of fatigue crack growth measurements are shown and discussed. The corresponding experimental conditions and the materials used are described in Section 3. A conclusion from the results is presented in Section 4.

## 2. Results and Discussion

### 2.1. Sample Geometry

**Figure 1:**
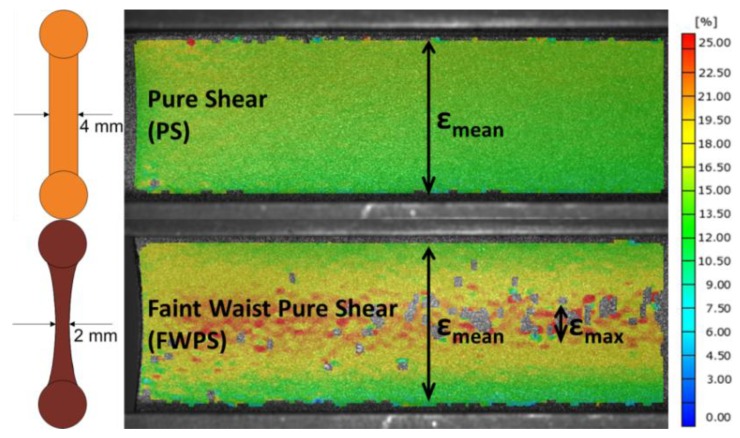
Strain distribution for flat pure shear (PS) and faint waist pure shear (FWPS) samples at equal displacement and corresponding cross-sections.

Two different sample geometries were investigated: one is a flat pure shear (PS) geometry with a thickness of 4 mm, and the other one is a so-called “faint waist pure shear” (FWPS) geometry with a nominal thickness of 2 mm. In [5], this investigation has been performed for an engineering material. Now, this investigation is resumed for a model material. The corresponding cross-sections can be found in Figure 1. It further shows the major strain distributions for the two geometries at equal displacement evaluated with 2D digital image correlation. As is also described in [5], the strain distribution is uniform for flat PS geometry, whereas it shows enhanced strain in the middle of the sample for FWPS geometry. Thus, the strain is constant along the height of the specimen for PS samples and equals a mean strain value, ε_mean_. For FWPS geometry, two strain values can be evaluated: a mean strain value averaged along the height, ε_mean_, and an enhanced strain, ε_max_.

**Figure 2:**
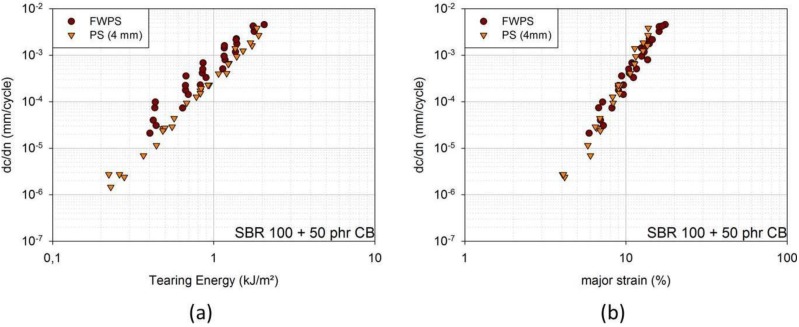
(**a**) Fatigue crack growth (FCG) curves in dependence of tearing energy for FWPS and PS samples [SBR 100 filled with 50 phr carbon black (CB)]; (**b**) FCG curves in dependence of major strain for FWPS and PS samples [SBR 100 filled with 50 phr carbon black (CB)].

In Figure 2a, FCG curves of an SBR blend with 50 phr (parts per hundred parts rubber) carbon black can be found in dependence of the tearing energy for the two geometries. The crack growth rates for the FWPS geometry are larger than for the PS geometry. Yet, the crack growth curves of the two overlap if crack growth rates are plotted against the major strain and if the enhanced strain, ε_max_, is taken into account for FWPS geometry (Figure 2b). The crack is affected by ε_max_; however, the tearing energy evaluated from force-displacement curves does not account for this enhanced strain, but is related to the mean strain, ε_mean_ [5].

One disadvantage of PS samples is that the crack sometimes grows out of the plane of the notch, especially at higher tearing energies. An additional manual evaluation of the crack images is needed to obtain the actual crack length. This is undesirable, because an additional evaluation increases the user effort. Crack growth out of the plane of the notch was mainly observed with the engineering blend [5] and seldom with the SBR blend. The major advantage of the PS geometry is that the tearing energy concept was developed for flat pure shear geometries [2,5]. Besides, the thicker PS geometry is more suitable for supporting tensional-compressional loads without buckling, which are of major interest in this study. Thus, PS geometry is used for the investigations described in the following subsections.

### 2.2. Frequency

To determine the influence of frequency, the maximum and minimum amplitude were kept constant, and the frequency and, therefore, the rate of strain were changed. Three frequencies were chosen as 1 Hz, 3 Hz and 5 Hz. These cover the frequency range that is of interest for handrails on test facilities.

**Figure 3:**
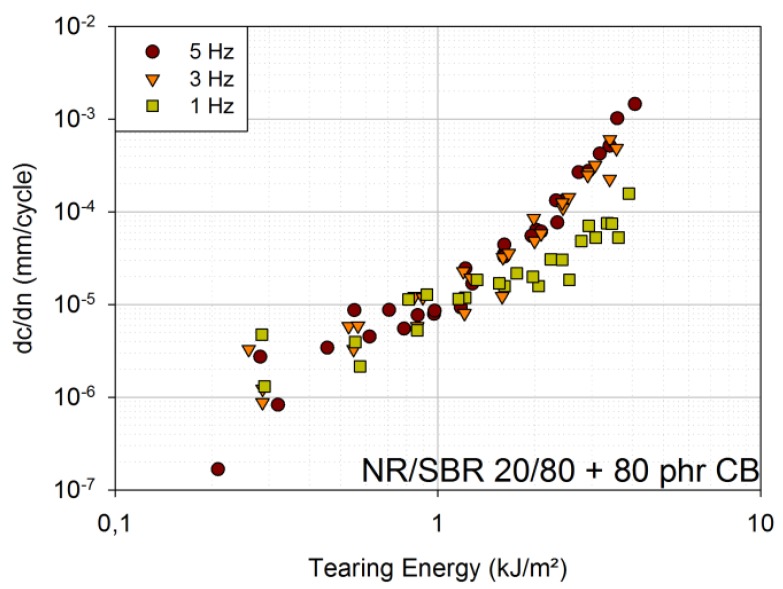
FCG curves for 1 Hz, 3 Hz and 5 Hz [engineering material: NR/SBR 20/80 filled with 80 phr carbon black (CB)].

Figure 3 shows the results for the engineering material (NR/SBR 20/80 with 80 phr carbon black). Up to tearing energies of approximately 1.5 kJ/m², no differences can be identified between the FCG curves of 1 Hz, 3 Hz and 5 Hz. Above approximately 1.5 kJ/m², the curves of 3 Hz and 5 Hz still overlap, whereas the curves of 1 Hz lie at lower crack growth rates.

**Figure 4:**
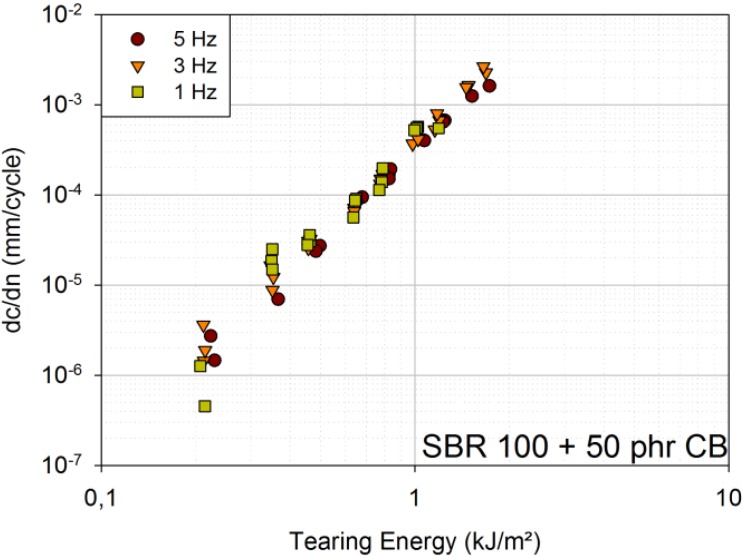
FCG curves for 1 Hz, 3 Hz and 5 Hz [model material: SBR 100 filled with 50 phr carbon black (CB)].

For the SBR blend with 50 phr carbon black, the curves for 1 Hz, 3 Hz and 5 Hz overlap over the whole tearing energy range tested (Figure 4).

Upon the first impression, these results are inconsistent with the literature. In [6,8], it is stated that amorphous rubbers, like SBR, show a very large influence of frequency, whereas rubbers that can strain-crystallize, like NR, show no influence of frequency. For amorphous rubbers, the dependency on frequency is attributed to time-dependent steady crack growth, which is inhibited by strain-induced crystallization (NR). However, it is also stated that time-dependent steady crack growth is mainly critical for frequencies below 0.2 Hz. As the lowest frequency in this study is 1 Hz, time-dependent crack growth should not be significant. Besides, time-dependent crack growth is substantially reduced by the addition of fillers, like carbon black [6].

It can be concluded that time-dependent crack growth, and, hence, frequency dependence, is smaller in filled amorphous rubbers than in unfilled ones. Nevertheless, time-dependent crack growth is significant mainly for very small frequencies. The FCG curves for different frequencies overlap, because the SBR blend is filled with 50 phr carbon black, and only frequencies down to 1 Hz are investigated. The same is true for the engineering material, but at tearing energies above 1.5 kJ/m², another effect seems to become more important: heat build-up due to cyclic loads. At 1.5 kJ/m², the plateau temperature is approximately 39 °C for 3 Hz and even approximately 47 °C for 5 Hz, whereas it is only approximately 28 °C for 1 Hz (tested under laboratory conditions). This large heat build-up represents another failure mechanism, and thermal failure is assumed to be added to the present mechanical failure [8].

For SBR with 50 phr carbon black, the temperature rise is far smaller (at 1.5 kJ/m² and 5 Hz only approximately 35.5 °C), due to the lower level of carbon black filler content. Samples of SBR with 50 phr carbon black have not been loaded up to the same tearing energies as the engineering material, due to far larger crack growth rates, even at small loads.

### 2.3. Ratio

The amplitude ratio equates to the minimum amplitude divided by the maximum amplitude and is therefore dimensionless. The investigation of the NR/SBR blend with 80 phr carbon black is of major importance, because it represents an actual engineering material. Thus, five different ratios were chosen for the engineering material [ratio −1; ratio −0.5; ratio 0; ratio +0.1; ratio +0.5]. For the two model materials (SBR with 50 phr carbon black and BR/SBR 50/50 with 50 phr carbon black), three ratios were investigated [ratio −0.5; ratio 0; ratio +0.5].

**Figure 5:**
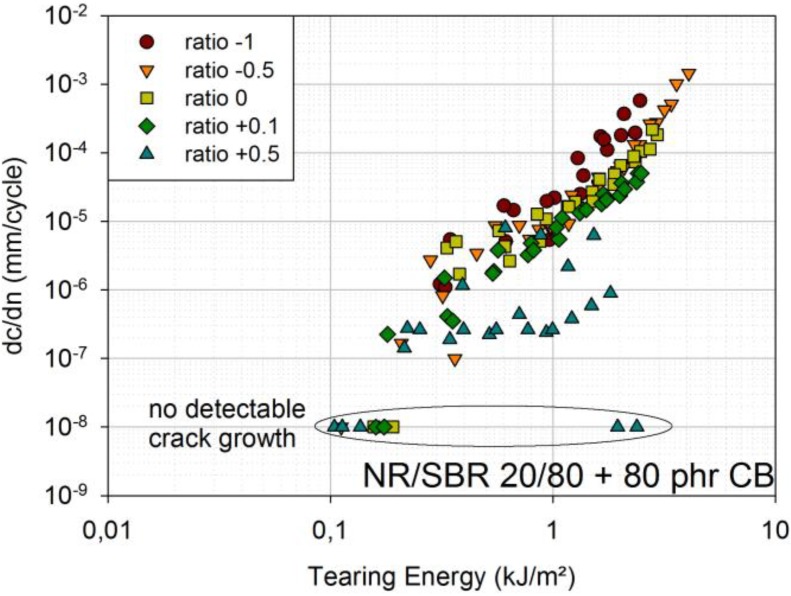
FCG curves for ratio −1; ratio −0.5; ratio 0; ratio +0.1; and ratio +0.5 [engineering material: NR/SBR 20/80 filled with 80 phr carbon black (CB)]; a non-detectable crack growth corresponds to a crack growth rate of 10^−8^ mm/cycle.

The results for the NR/SBR blend are presented in Figure 5. Compared to ratio 0, FCG curves for ratio +0.1 lie at slightly smaller crack growth rates, and for ratio +0.5, they lie at significantly smaller crack growth rates. The measurements with ratio +0.5 show quite a large scatter, because sometimes, no crack growth was detectable at all. In order to display such points in a double-logarithmic diagram, they were associated with a crack growth rate of 10^−8^ mm/cycle.

Compared to ratio 0, ratio −0.5 shows similar crack growth rates; only at higher tearing energies, the crack growth rates of −0.5 are slightly larger. This can be attributed to the fact that there is a small compressional region, even for ratio 0, due to the permanent set [11].

The FCG curves for ratio −1 clearly lie at the largest crack growth rates. This is also described in the literature [12], where increased fatigue crack growth for ratio −1 is associated with a chemical degradation at the crack tip caused by large compressional loads.

**Figure 6:**
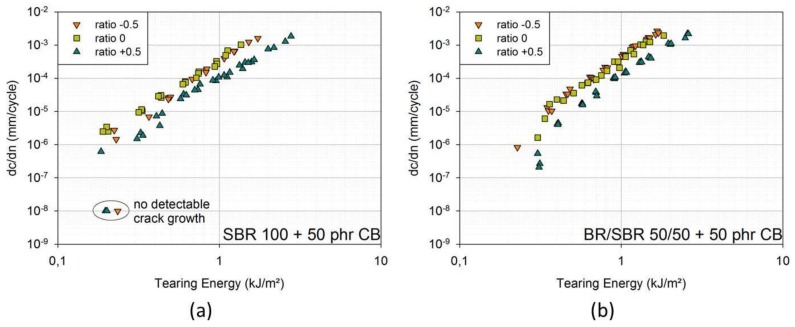
(**a**) FCG curves for ratio −0.5; ratio 0; ratio +0.5; (model material: SBR 100 filled with 50 phr carbon black (CB)); (**b**) FCG curves for ratio +0.5; ratio 0; ratio −0.5; (model material: BR/SBR 50/50 filled with 50 phr carbon black (CB)). Non-detectable crack growth corresponds to a crack growth rate of 10^−8^ mm/cycle.

In Figure 6a,b, one can see the results for SBR with 50 phr carbon black and BR/SBR 50/50 with 50 phr carbon black. In both cases, FCG curves for ratio +0.5 lie at significantly smaller crack growth rates. FCG curves for ratio 0 and −0.5 overlap. For BR/SBR, the curves for ratio 0 lie at slightly smaller crack growth rates than for ratio −0.5, though the differences are small.

All results indicate a beneficial effect of a positive loading ratio. Usually, this effect is attributed to strain-crystallizing rubbers [6]. However, the beneficial effect of an increased minimum strain has been also described in [13] for filler reinforced non-strain-crystallizing rubbers and is attributed to the filler-polymer system.

### 2.4. Waveform

In this study, three different types of loading waveforms were investigated. They were chosen in a way so that the frequency (time for a whole loading cycle) is constantly 1 Hz.

**Figure 7:**
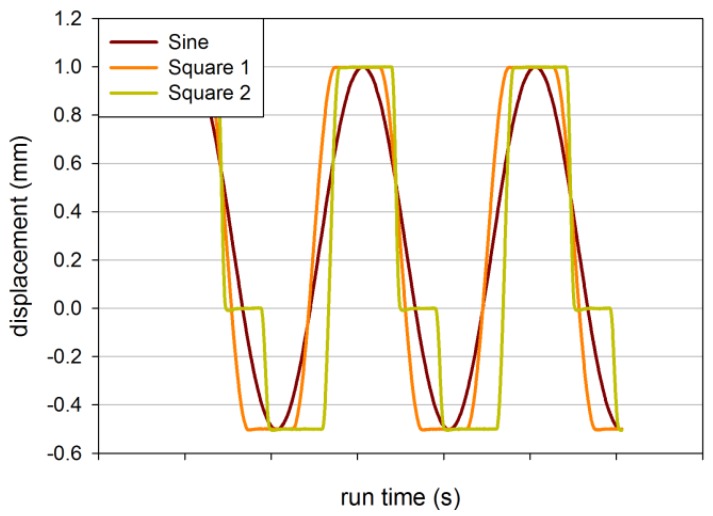
Displacement *versus* run time for different waveform types: “sine”, “square 1” (square with sinusoidal slopes) and “square 2” (square with sinusoidal slopes with longer dwell times).

In Figure 7, the measured run time displacement data are shown for the three waveforms in order to illustrate their shape. The first one, further called “sine”, is a usual sinusoidal loading, which serves as the reference, because sinusoidal loads have been sufficiently investigated. The second one, further called “square 1”, is a mixture of a square loading shape and a sinusoidal loading. Its major shape is square, but its slopes are sinusoidal. The third waveform, further called “square 2”, is a mixture of a square loading shape and a sinusoidal loading, as well, but the dwell periods at maximum and minimum amplitude are increased and another dwell period at zero amplitude is added. Consequently, the sinusoidal slopes get steeper.

**Figure 8:**
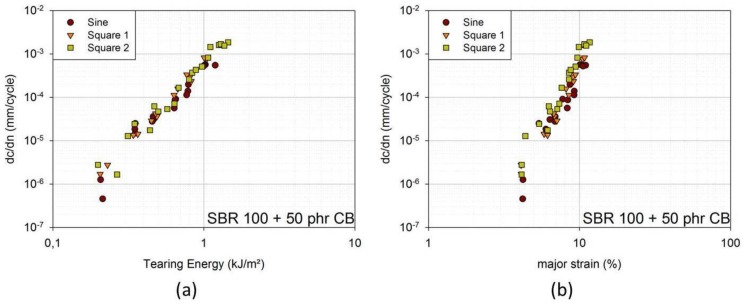
(**a**) FCG curves in dependence of tearing energy for waveform “sine”, “square 1” and “square 2” (model material: SBR 100 filled with 50 phr carbon black (CB)); (**b**) FCG curves in dependence of major strain for waveform “sine”, “square 1” and “square 2” [model material: SBR 100 filled with 50 phr carbon black (CB)].

If dwell periods are added (“square 1” and “square 2”), the evaluation of tearing energy was performed as with sinusoidal loading (compare Section 3.1.), whereas the change in force during dwell time is not taken into account. Thus, the evaluation corresponds to the evaluation of sinusoidal loading conditions as much as possible. Yet, FCG curves are additionally plotted against major strain to account for discrepancies in the evaluation. In Figure 8a,b, the FCG curves for SBR with 50 phr carbon black are shown in dependence of the tearing energy and on the major strain. In dependence of major strain, the curves for “square 1” lie at higher crack growth rates than the ones for “sine”. The curves for “square 2” lie at the highest crack growth rates.

Keeping in mind the results obtained in Section 2.2. (no influence of strain rate on FCG curves for SBR with 50 phr carbon black), it seems as if dwell periods are the major factors that lead to increased crack growth rates for “square 1” and “square 2” compared to “sine”. There are longer dwell periods in “square 2” than in “square 1”, and “square 2” also includes a zero-amplitude dwell period. Crack growth rates are lager for “square 2” accordingly.

In [14], it is stated that an increase in dwell time and mainly dwell strain levels near zero-stress both result in higher crack growth rates, especially for SBR. This is attributed to time-dependent recovery in the rubber microstructure in the vicinity of the crack tip, which results in an elevated stress-state.

### 2.5. Heat Build-Up

According to [6], the heat generation per second during cyclic loading is given by the dissipated energy per cycle, times the frequency. For the current investigation, the heat generation is divided by the cross-section area of the sample in order to take into account sample geometry. Thus, it is suggested to display resulting plateau temperatures in dependence of dissipated energy per time and per unit area.

**Figure 9:**
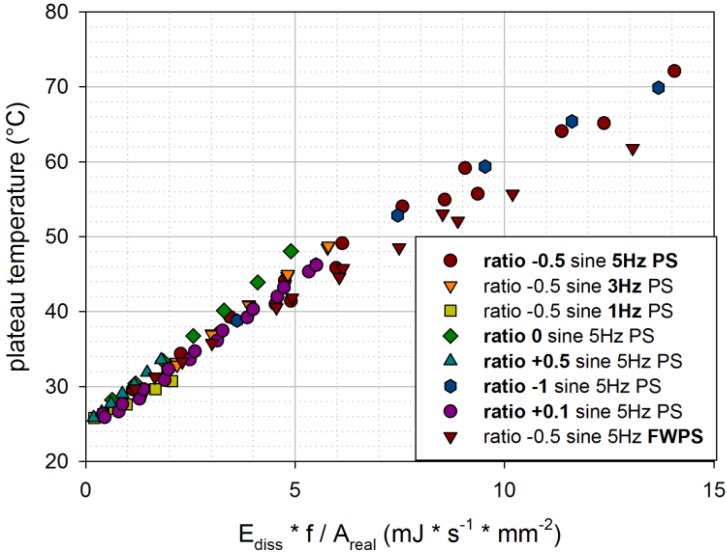
Plateau temperatures for various loading conditions in dependence of the dissipated energy per second and per unit area [engineering material: NR/SBR 20/80 filled with 80 phr carbon black (CB)].

In Figure 9, plateau temperatures for the engineering material (NR/SBR 20/80 with 80 phr carbon black) are plotted against dissipated energy per second and per unit area. Plateau temperatures are obtained for various loading conditions (various amplitude ratios, frequencies and sample geometries). Though there is quite a large scatter, a linear trend can be observed. At higher tearing energies, plateau temperatures of FWPS are lower compared to PS, probably because of buckling during compression.

For SBR with 50 phr carbon black and BR/SBR 50/50 with 50 phr carbon black, the results from fatigue crack growth measurements cannot be used to determine plateau temperatures, because the crack growth is very large, and therefore, the number of cycles was substantially reduced. Thus, measurements of plateau temperatures were performed separately on non-cut samples.

Approximately linear relationships can be observed also for SBR with 50 phr carbon black and BR/SBR 50/50 with 50 phr carbon black for the variation of frequency and amplitude ratio. Though, especially for SBR with 50 phr carbon black, there is quite a large scatter.

**Figure 10:**
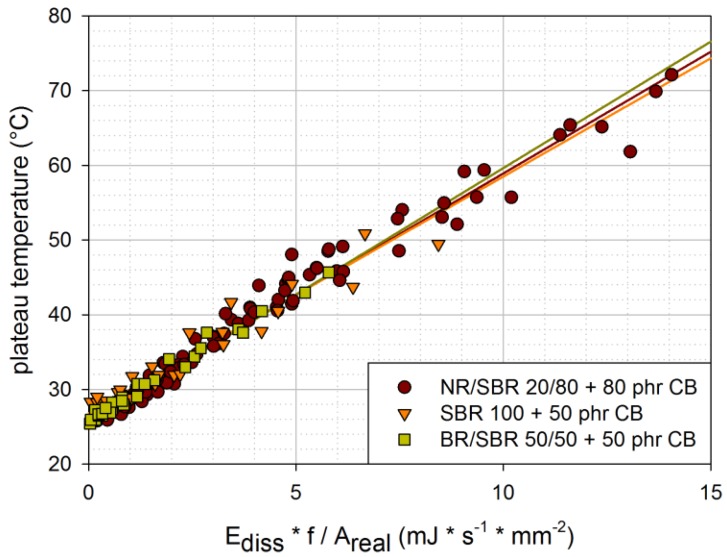
Plateau temperatures for various loading conditions in dependence of the dissipated energy per second and per unit area [engineering material: NR/SBR 20/80 filled with 80 phr carbon black; model material: SBR 100 filled with 50 phr carbon black; model material: BR/SBR 50/50 filled with 50 phr carbon black (CB)].

In Figure 10, the plateau temperatures determined under various loading conditions are plotted for the three investigated materials. The linear fits nearly overlap for all three materials. The differences in material behavior seem to be covered by the dissipated energy, which is determined from force-displacement curves. Furthermore, all investigated rubber blends seem to have similar coefficients of thermal conduction. This is in accordance with [6], where it is stated that thermal conductivity in rubber is fairly independent of the elastomer.

Nevertheless, it has to be emphasized that the scatter of the results in Figure 10 is quite large. Thus, the dissipated energy per time and unit area only gives an idea of the plateau temperature reached. There are probably other influence factors that are not considered in this very simple relationship.

In order to further test this quite simple relationship, additional dynamic mechanical analysis measurements were performed. Temperature differences were calculated from the plateau temperature and the sample temperature before loading. Very few measurements led to evaluable data, as sometimes, the sample shifted within the fixtures or the resulting loads exceeded the load limit. For BR/SBR 50/50 filled with 50 phr carbon black, only two measurements were evaluable.

However, the temperature differences in dependence of dissipated energy per second and per unit area show, again, a linear relationship. Yet, it has to be emphasized that the heat build-up within the sample is very small compared to the results of usual fatigue measurements. This can be attributed to the far smaller sample size and the lower loads applied.

## 3. Experimental Section 

### 3.1. Fatigue Crack Growth Measurement

In order to determine FCG curves, it is necessary to measure fatigue crack growth rate at certain loading conditions. These loading conditions are characterized by the fracture mechanical parameter of the tearing energy. The fracture mechanical concept of the tearing energy was developed by Rivlin and Thomas, and it was later extended from quasistatic to cyclic loading conditions [2,3,4].

The fatigue measurement procedure applied in this paper consists of several loading steps with constant frequency, a constant amplitude ratio and increasing maximum amplitude. If not stated differently, the amplitude ratio is −0.5, the frequency 5 Hz and the waveform sinusoidal. For model materials, the maximum amplitudes correspond to strains in the range of 4% to 20% and for the engineering material in the range of 2.5% to 36%. The minimum amplitude results from the amplitude ratio.

Each loading step consists of preconditioning cycles and measurement cycles [15]. The crack growth is calculated from the crack length before and after the measurement cycles. The preconditioning cycles are necessary in order to neglect the larger initial crack growth rate and the corresponding roughening of the crack tip [16,17].

In this study, the tearing energy for each loading step is determined dynamically, which means during the measurement, at the end of the measurement cycles. For pure shear samples, the tearing energy is given by Formula 1, in which the strain energy, U, represents the area under the force-displacement curve.

(1) $$T=W_{0}\;\cdot \;h_{0}=\frac{U}{A_{uncracked}}$$

If the minimum force is smaller than zero (for amplitude ratios smaller or equal to zero), the strain energy is determined by Formula 2 [18]. In this case, the tensional area under the force-displacement curve is considered. If the minimum force is greater than zero, the strain energy consists of two parts that are additive [19]. The first one, *U*_max_, is the area under the force-displacement curve ranging from minimum force to maximum force. The other one, *U*_min_, is the strain energy at minimum strain, which is approximated by multiplying the minimum force with the displacement at minimum force. Formula 3 shows the determination of strain energy for positive amplitude ratios (based on [19]).

(2) U=∫(F|F≥0)ds

(3) $$U=U_{\text{min}}+U_{\text{max}}=F_{\text{min}}\;\cdot \;ds\left(F_{\text{min}}\right)+\;\int_{F_{\text{min}}}^{F_{\text{max}}}F\;\cdot \;\text{d}s$$

The measurement setup is composed of a dynamic testing system, MTS 858 MiniBionix (MTS Systems Corporation), and an optical measurement and evaluation system, CV 5001 Series (Keyence). The measurement setup has been described in detail in [5,15]. One special feature is that not only shadow images, but also fully illuminated images of the crack are taken. This provides the advantage that deviations of crack shape and growth can be detected and considered in the evaluation. With that, the reproducibility of the FCG curves is improved, and their trend gets smoother [5]. Another special feature is that a swivel arm permits us to take additional pictures from the back side of the sample. They are obtained at zero and maximum displacement of each loading step and give the possibility of determining strain distribution by subsequent 2-dimensional full field strain analysis with the digital image correlation software Aramis (GOM mbH - Gesellschaft für Optische Messtechnik) [5,20].

Due to internal frictional forces, heat is generated under cyclic loads. The temperature of the rubber article rises until heat generation is balanced by heat transfer to the surroundings [9,21]. A constant temperature is reached, which will be further called plateau temperature. To measure the heat build-up during fatigue, a complementary thermal imaging system, FLIR ThermoVision A20 (FLIR Systems, Inc.), is used. It is equipped with an uncooled microbolometer to measure infrared radiation (heat) and it has a thermal sensitivity of 0.12 °C at 30 °C. The emissivity was adjusted to 0.92, according to [22]. The emissivity can be assumed to be independent of deformation and, therefore, constant throughout the tests [23].

The data acquisition rate is adapted, so that precise temperature curves are obtained and the amount of data is still reasonable. The dissipated energy is calculated from force-displacement curves and represents the area of the hysteresis loop.

### 3.2. Dynamic Mechanical Analysis (DMA)

Dynamic mechanical analysis (DMA) measurements were performed using the DMA system RSA G2 (TA Instruments). Smaller samples were cut from the usual pure shear samples, so that the load limit is not exceeded. Their thickness is 4 mm, and their width is approximately 7 to 9 mm. The clamping distance is approximately 11 mm, so that the load amplitude does not exceed the machine limits. The material`s characteristics, like storage modulus, loss modulus and tangent delta, were saved during loading, as well as the ambient temperature. To determine the heat build-up in the sample, a thermocouple was used. It was introduced to the middle of the sample through a small hole pinned with a very thin needle. The temperature was plotted with a data logger. Measurement conditions included three environmental temperatures (0 °C, 25 °C and 50 °C) and two strain levels (5% strain and 10% strain). The test frequency was 10 Hz.

### 3.3. Materials

Three different materials were used in this study. One is an engineering material that is used in actual products. The other two are model materials that contain as little ingredients as possible. The basic formulations are given in Table 1. For propriety reasons, no further information about the exact formulations can be given.

**Table 1:** Basic formulations of investigated materials.

Ingredient	Engineering material (phr)	Model material 1 (phr)	Model material 2 (phr)
SBR	80	100	50
BR	–	–	50
NR	20	–	–
Carbon black	80	50	50

## 4. Conclusions

The results show that for filled rubber blends, frequency is not that important in the investigated range. However, if the heat build-up exceeds a certain limit, due to large filler content and/or frequencies, the influence of frequency on fatigue crack growth can be observed.

Further, it was shown that the amplitude ratio has a big influence on fatigue. Especially if the minimum strain is tensional (positive loading ratios), the crack growth is reduced. This effect has been observed not only for a blend with strain-crystallizing NR, but also for non-strain-crystallizing rubber blends. The opposite effect was observed for negative amplitude ratios, *i.e.*, for tension-compression loading. For those ratios, the crack growth was considerably increased.

Furthermore, the results indicate that also the waveform of the loading is very important. Taking into account the independence of strain rate (frequency), it can be concluded that the primary influence factors are the dwell periods. If dwell periods are added to the sinusoidal waveform, crack growth is increased (compare “sine” and “square 1”). If the dwell periods at maximum and minimum displacement are extended and a dwell period with a zero-amplitude level is added, the crack growth rate is further increased (compare “square 1” and “square 2”).

The investigations show that the proximity to reality is of major importance. Especially dwell periods should not be neglected, and the waveform should be adapted to take them into account. Further, the fatigue behavior of rubber is influenced by the amplitude ratio. There are large differences in crack growth rates, whether the loads are tensional and compressional or purely tensional. The beneficial effect of positive loading ratios can be attributed to strain-crystallization and the filler-polymer system [6,13]. On the contrary, the degradation at the crack tip in the case of compressional loads has a deleterious effect on fatigue crack growth [6,12].

Plateau temperatures are determined for various loading conditions and two sample geometries. For all investigated materials, they show a similar linear relationship in dependence of dissipated energy per second and unit area. Although the results show fairly large scatter, this simple relationship can be used to give an idea of the expected plateau temperatures.

The results obtained by dynamic mechanical analysis show too little heat build-up to compare them to the ones of fatigue measurements. Thus, heat build-up at different environmental temperatures should be further investigated with fatigue measurements using a temperature chamber.
